# Choice of Commercial DNA Extraction Method Does Not Affect 16S Sequencing Outcomes in Cloacal Swabs

**DOI:** 10.3390/ani11051372

**Published:** 2021-05-12

**Authors:** Emily Van Syoc, Natália Carrillo Gaeta, Erika Ganda

**Affiliations:** 1 Integrative & Biomedical Physiology and Clinical & Translational Sciences Dual-Title Ph.D. Program, Pennsylvania State University, University Park, PA 16802, USA; epb5360@psu.edu; 2 Department of Animal Science, College of Agricultural Sciences, Pennsylvania State University, University Park, PA 16801, USA; 3 Department of Internal Medicine, School of Veterinary Medicine and Animal Science, University of Sao Paulo, Sao Paulo 05508-270, Brazil; natalia.gaeta@hotmail.com

**Keywords:** poultry microbiome, cloacal microbiome, animal microbiome, method evaluation

## Abstract

**Simple Summary:**

The cloacal anatomy is unique because the fecal, urinary, and reproductive tracts converge into one orifice. Therefore, sampling for microbiome research can be difficult in birds, especially in agricultural production settings where it may not be feasible to sample the intestines, and cloacal swabs are often used. There is a need to evaluate laboratory methods for 16S rRNA sequencing in cloacal swab samples to ensure reproducible and trustworthy downstream results. We compared four DNA extraction methods from two commercially available magnetic-based DNA extraction kits. Mock communities and negative controls were included for each method and subjected to 16S rRNA sequencing. While extraction quality and yield differed between each extraction method, overall sequencing results were not affected, including alpha and beta diversity. Positive and negative controls are an important aspect of microbiome science and our findings lend guidance to future microbiome research in poultry.

**Abstract:**

As the applications of microbiome science in agriculture expand, laboratory methods should be constantly evaluated to ensure optimization and reliability of downstream results. Most animal microbiome research uses fecal samples or rectal swabs for profiling the gut bacterial community; however, in birds, this is difficult given the unique anatomy of the cloaca where the fecal, urinary, and reproductive tracts converge into one orifice. Therefore, avian gut microbiomes are usually sampled from cloacal swabs, creating a need to evaluate sample preparation methods to optimize 16S sequencing. We compared four different DNA extraction methods from two commercially available kits on cloacal swabs from 10 adult commercial laying hens and included mock communities and negative controls, which were then subjected to 16S rRNA amplicon sequencing. Extracted DNA yield and quality, diversity analyses, and contaminants were assessed. Differences in DNA quality and quantity were observed, and all methods needed further purification for optimal sequencing, suggesting contaminants due to cloacal contents, method reagents, and/or environmental factors. However, no differences were observed in alpha or beta diversity between methods. Importantly, multiple bacterial contaminants were detected in each mock community and negative control, indicating the prevalence of laboratory and handling contamination as well as method-specific reagent contamination. We found that although the extraction methods resulted in different extraction quality and yield, overall sequencing results were not affected, and we did not identify any method that would be an inappropriate choice in extracting DNA from cloacal swabs for 16S rRNA sequencing. Overall, our results highlight the need for careful consideration of positive and negative controls in addition to DNA isolation method and lend guidance to future microbiome research in poultry.

## 1. Introduction

Rapidly advancing technology has made amplicon sequencing a popular and readily available method for analyzing microbiomes. As the field of microbiome science expands, so too does the need to standardize and optimize laboratory methods for best sequencing outcomes. There is growing interest in manipulating animal microbiomes to optimize production agricultural outcomes [1]. For example, recent studies in poultry production have focused on alternatives to antibiotic feed supplementation to improve health and mitigate disease [2,3,4], and work in poultry microbiomes is expanding [5]. The cloacal anatomy makes traditional fecal sampling difficult as the urogenital, reproductive, and fecal tracts all converge at the cloacal opening. Thus, where other animals may be sampled by swabbing the rectum for a fresh fecal sample, swabbing the cloaca of birds is known to include bacterial communities of these different organ tracts [6,7]. Therefore, validation in laboratory methods performed on fecal samples cannot necessarily be extrapolated to cloacal samples. As such, commercial laying hens were chosen for this study to explore whether the choice of commercial DNA method affects library preparation or sequencing results.

The first step of 16S sequencing library preparation is to extract genomic DNA from the sample. Today, this is commonly performed with a commercial DNA extraction method that includes proprietary reagents and methodology. Past work has suggested that the choice of commercial DNA extraction method can affect DNA yield and quality [8,9] or even microbial diversity metrics [10,11]. However, these results seem to be system-specific, and to our knowledge, no previous work has been done to optimize DNA extraction from cloacal swab samples. Sequencing technology has vastly accelerated over the last 10 years, and it is important to continually evaluate laboratory methods to ensure that downstream results are reproducible and trustworthy.

To address this knowledge gap, we compared four different extraction protocols from two commercially available magnetic-based DNA extraction kits on cloacal swab samples collected from 10 hens. Positive and negative controls for each method were extracted and sequenced alongside the samples to enable comparison of method-specific contaminants. Extracted DNA quality and quantity were measured prior to library preparation and 16S sequencing. These data provide guidance towards appropriate methods for extraction of DNA from cloacal swabs for best 16S sequencing results in cloacal samples.

## 2. Materials and Methods

### 2.1. Sample Collection

All animal procedures described in this manuscript were approved by the Pennsylvania State University’s Institutional Animal Care and Use Committee (protocol no. PRAMS201647461). Female White Leghorn chickens (Hy-Line W36 strain) were reared in individual cages at the Poultry Education and Research Center of the Pennsylvania State University and exposed to a photoperiod of 16 h light and 8 h dark in a ventilated layer house kept at a standard 25–26 °C temperature (University Park, PA, USA). The animals were provided with layer mash diet and water ad libitum. At the time of sampling, the hens were approximately 2.8 years old. The animals were randomly subset from an ongoing study on metformin supplementation. Egg production records were maintained daily to determine ovulatory cycle patterns. Four cloacal samples from 10 adult White Leghorn chickens (totaling 40 samples) were collected using a sterile swab (double package, Puritan, Guilford, ME, USA) by inserting the swab ~1 inch into the cloaca and swirling for 2–3 s. All four swabs were introduced at once in the cloaca and swirled together, and all samples were collected on the same day. The swabs were immediately placed into sterile microtubes, transported to the laboratory on ice, and stored at −80 °C until DNA extraction.

### 2.2. DNA Extraction

Before DNA extraction, all swab samples were randomized and submitted to a pre-processing step. Briefly, one milliliter of sterile phosphate buffer saline solution (PBS; 1x, pH = 7.4) was added to each tube, which were homogenized at 20 Hz for 30 min in the Bead-Ruptor96 (Omni International, Kennesaw, USA). Samples were then transferred to a new tube and centrifuged at 14,000× *g* for 30 min to pellet bacterial cells and debris. Finally, each pellet was suspended in 300 µL of PBS and homogenized again for 30 min in the same conditions described above. All samples were kept at −80 °C until processing.

DNA extraction was performed in a UV-sterilized biosafety cabinet, and pipettes and other equipment were sterilized with 70% ethanol in between each extraction. Genomic DNA was extracted from cloacal samples, a mock community (Zymo Research, Irvine, CA, USA), and negative controls (buffers) using two different DNA extraction methods and four different protocols: MagMAX™ CORE mechanical lysis module and MagMAX™ CORE nucleic acid purification method (Referred to as Method 1; ThermoFisher Scientific, Waltham, MA, USA), MagMAX™ microbiome ultra nucleic acid isolation method (Referred to as Method 2; ThermoFisher Scientific, Waltham, MA, USA), in-house bead loading system and MagMAX™ CORE nucleic acid purification method using clarifying solution (Referred to as Method 3; ThermoFisher Scientific, Waltham, MA, USA), and in-house bead loading system and MagMAX™ CORE nucleic acid purification method using Reagent DX (Referred to as Method 4; ThermoFisher Scientific, Waltham, MA, USA). For Method 1 and Method 2, DNA extraction was performed according to the manufacturer’s instructions. For Method 3 and Method 4, DNA extraction was performed according to the manufacturer’s instructions, except for the use of zirconia bead tubes (Biospec, Bartlesville, OK, USA), within which samples were lysed before they were added to the sample plate. Briefly, 450 μL of lysis buffer were added to each zirconia bead tube corresponding to a sample. The lysis solution was prepared with the lysis buffer included in the methods and 2.05 μL/reaction of clarifying solution (ThermoFisher Scientific, Waltham, MA, USA) or Reagent DX (Qiagen Inc., Hilden, Germany) for Method 3 and Method 4 protocols, respectively. Subsequently, 200 μL of each sample were transferred to the zirconia tubes, which were homogenized in the Bead-Ruptor96 (Omni International, Kennesaw, GA, USA) at 20 Hz for 2.5 min. Samples rested for 5 min and were homogenized for an additional 2.5 min. Zirconia tubes were then centrifuged at 2500 rpm for five minutes, and 500 μL of the lysate from zirconia tubes were transferred to the sample plate, and each protocol proceeded according to the manufacturer’s instructions.

Extracted DNA quantity was measured fluorometrically with Qubit (ThermoFisher Scientific, Waltham, MA, USA) at the Pennsylvania State University Genomics Core Facility and spectrophotometrically with a NanoDrop (ThermoFisher Scientific, Waltham, MA, USA). The extracted DNA was stored at −80 °C until further library preparation.

### 2.3. 16S Library Preparation

Polymerase chain reaction was performed on genomic DNA with the standard 515F and 816R primers- FWD: GTG**Y**CAGCMGCCGCGGTAA; REV: GGACTAC**N**VGGGTWTCTAAT, following Earth Microbiome Project protocols [12,13]. The PCR products were checked on a 1% agarose gel and cleaned with AmPure magnetic beads (Beckman Coulter, Brea, CA, USA) following manufacturer’s instructions. The quality and quantity of cleaned DNA was assessed with a spectrophotometer (NanoDrop). Amplicons were transported on ice to the Genome Sciences and Bioinformatics Core of the Penn State College of Medicine, where they were indexed with Illumina barcodes, library was prepared, checked for quality, and 16S sequencing was performed with an Illumina MiSeq at 2 × 300 bp using V3 chemistry.

### 2.4. 16S Taxonomic Assignment

Reads were processed using the dada2 (v. 1.18.0) R package (Callahan et al. 2016). Raw reads were quality-filtered using the “filterAndTrim” option. Twelve different parameters were tested to optimize the quality-filtered reads (Table S1) based on the plot quality profile. Best results were obtained using the following parameters (truncLen = c (275, 280), trimLeft = c (20, 20), maxN = 0, maxEE = c (2, 2), truncQ = 2, rm.phix = TRUE, compress = TRUE, multithread = FALSE), which were used in the data analysis. Chimeras were identified using the “removeBimeraDenovo” tool and were removed from the data. Taxonomic assignment was performed using the “assignTaxonomy” tool with the Silva database v.138 (https://www.arb-silva.de/documentation/release-1381/, accessed on 1 February 2021). Data were rarefied based on the sample with the lowest sequence count using the “rarefy_even_depth” tool of the R package phyloseq v 1.34.0 and used for diversity analysis [14]. Data were normalized to ASV count in which ASV counts were divided by the total ASV and used for relative abundance.

### 2.5. Statistical Analyses

As the animals sampled were a subset from a larger study where they were supplemented with medicated feed, the data were assessed to ensure the data from individual chickens were alike enough to be considered biological replicates. Data from all four methods were combined and Kruskal–Wallis was performed for the variables of DNA quantity (ng/µL), A260/280, and A260/230 ratios between chickens. No significant difference was found, so individual chickens were treated as replicates. All variables were assessed for normality by plotting a histogram and performing Shapiro–Wilks’s test [15], following which non-parametric tests were used throughout. Positive and negative controls were not included in the tests between DNA extraction methods. Differences between the methods in DNA extraction yield (quantity, as measured with Qubit) were tested using the Kruskal–Wallis test, and post hoc comparisons were made with Dunn’s test in the FSA R package [16]. Two commonly used spectrophotometric ratios, as measured with NanoDrop, were calculated to assess the purity of extracted DNA: A260/280 and A260/230. These ratios were then scaled to represent the difference from the “ideal” purity value. The ideal values were 1.80 and 2.00 for A260/280 and A260/230, respectively [17]. To determine whether the median difference from the ideal value for each method was significantly different from 0, a two-sided Wilcox rank sum test was performed with the null hypothesis set to 0.

Alpha diversity metrics (Shannon’s index and observed taxa) were calculated with the phyloseq package and visualized with the ggplot package [14,18]. Bray–Curtis and weighted Unifrac distances were calculated in the phyloseq package and visualized with non-metric multidimensional scaling and principal coordinate analysis. Alpha diversity indices were assessed for normality by plotting a histogram and performing Shapiro–Wilks’s test [15]. Shannon’s index and observed taxa were compared among different DNA extraction methods using Kruskal–Wallis test with post hoc comparisons using the Dunn test. The microbial community composition (beta diversity) was assessed for similar group dispersions with the ”permutest” and “betadisperser” functions prior to testing with permutational ANOVA using the “adonis” function in the vegan R package [19]. Taxonomic plots were generated to describe the most abundant phyla and genera in fecal samples, mock community, and negative controls. The relative abundance of phyla and genera was tested for differences between methods with Kruskal–Wallis test, after which *p* values were adjusted for multiple comparisons with Bonferroni’s correction. After adjustment, significant results were subjected to Dunn’s post hoc test. All statistical analyses were performed in R version 4.0.1, and raw data (NanoDrop and Qubit data) and R scripts are available at https://github.com/gandalab/bird-dna-extraction. Sequencing reads are available at the NCBI Sequence Read Archive under accession number PRJNA720437.

## 3. Results

### 3.1. DNA Quantity and Quality

**Figure 1:**
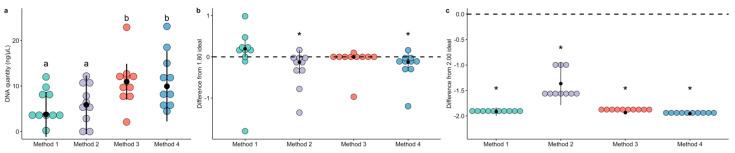
The quantity and quality of extracted DNA varies by commercial extraction method. DNA quantity was measured with a Qubit instrument and compared between methods (**a**, Dunn test *p* < 0.05, indicated with lowercase letters). DNA purity was assessed with NanoDrop and the standard ratios were transformed to reflect the differences from the ideal purity value. Each method was compared to the ideal value; the ideal for A260/280 is 1.80 (**b**, Wilcox rank sum test *p* < 0.05, indicated with asterisks) and the ideal value for A260/230 is 2.00 (**c**, Wilcox rank sum test *p* < 0.05, indicated with asterisks).

The quantity of extracted DNA, measured through fluorescence with a Qubit instrument, differed between extraction methods. Method 3 and Method 4 had higher extraction yield than both Method 1 and Method 2 (Figure 1a and Table 1). Specifically, Method 3 had higher DNA quantity than both Method 1 (Dunn’s test Z = −2.30, *p* = 0.021) and Method 2 (Dunn’s test Z = −2.19, *p* = 0.028), and Method 4 had higher DNA quantity than both Method 1 (Dunn’s test Z = −2.22, *p* = 0.026) and Method 2 (Dunn’s test Z = −2.11, *p* = 0.03). There were no differences between Method 3 and Method 4 (Dunn’s test Z = 0.08, *p* = 0.94) or between Method 1 and Method 2 (Dunn’s test Z = −0.11, *p* = 0.91).

The ratio of A260 to A280 absorbances (A260/280) are used to assess the purity extracted DNA as nucleic acids absorb at 260 nm and proteins absorb at 280 nm [17]. Median A260/280 values are reported in Table 1. We normalized the ratio data to difference from the “ideal” 1.80 ratio to assess whether any extraction method resulted in undesired protein contamination. Individual comparisons for each method revealed that Method 2 (Wilcox rank test V = 6.5, *p* = 0.037) and Method 4 (Wilcox rank test V = 7.0, *p* = 0.041) differed significantly from the ideal ratio, while Method 1 (Wilcox rank test V = 35.0, *p* = 0.155) and Method 3 (Wilcox rank test V = 24.0, *p* = 0.906) did not (Figure 1b).

The ratio of A260 to A230 absorbances (A260/230) are considered a broad category for detecting contaminants, including phenols. Median A260/230 values for each method are reported in Table 1. The data were normalized in the same way as the A260/280 ratios, and 2.0 was considered the “ideal” ratio [17]. Notably, all four methods were characterized by very low A260/230 ratios (Figure 1c). Method 1 (Wilcox rank test V = 0.0, *p* = 0.0056), Method 2 (Wilcox rank test V = 0.0, *p* = 0.0059), Method 3 (Wilcox rank test V = 0.0, *p* = 0.0059), and Method 4 (Wilcox rank test V = 0.0, *p* = 0.0058) all had strong differences from the ideal ratio. However, after PCR amplification and AmPure magnetic bead cleaning, the ratios were recovered to acceptable values before sequencing, and only Method 2 had significant differences from the ideal 2.00 value (Figure S1).

### 3.2. Cloacal Microbiome

Sequencing of the 16S rRNA V4 hypervariable region was carried out in 40 samples using the Illumina MiSeq platform. A total of 6,595,579 reads were obtained from cloacal swab samples, with a mean read count of 196,889 per sample and a mean read count of 1,648,895 per DNA extraction method (Method 1 = 1,721,845 reads; Method 2 = 1,521,348 reads; Method 3 = 1,643,154 reads; and Method 4 = 1,709,232 reads). Raw sequencing data were visualized in MultiQC to determine filtering and trimming parameters (Table S1). Filtering and trimming were performed in dada2 and the median value for input, filtered, merged, and non-chimeric reads did not differ among extraction methods (Table 2). The average number of non-chimeric reads in cloacal samples (*n* = 139,423) and positive controls (*n* = 129,971) were 6.34 and 5.91 times higher than negative controls (*n* = 21,979.5), respectively. The negative controls in all four methods had detectable bacterial biomass that was reflected in the respective counts of non-chimeric reads (Method 1 = 12,812; Method 2 = 4472; Method 3 = 19,819; Method 4 = 50,815).

### 3.3. Assessment of Mock Community and Negative Controls

**Figure 2:**
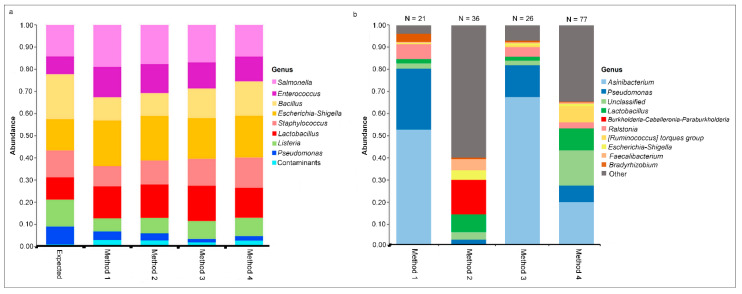
Distribution of genera detected in the mock community is similar across each DNA extraction method, but not among negative controls. Relative abundance of the genera detected in mock community (**a**) and negative controls (**b**) among each DNA extraction method. In the mock community, contaminants are considered any genera not among those specified by the expected standard. In the negative controls, the top 10 genera are shown in each method and the total relative abundance of additional genera are shown in the grey “Other” category. The number of total genera detected in each negative control is shown at the top of the bar chart.

To enable comparison of method-specific contaminants, a negative (extraction buffer blank) and positive (known mock community standard) control were extracted, amplified, and sequenced for each commercial magnetic-based extraction method. The mock community composition of each method was compared to the expected values given by the mock community standard manufacturer. All methods showed similar composition with minimal detected contaminants in the positive controls and recapitulated the expected composition of the mock community (Figure 2a).

**Figure 3:**
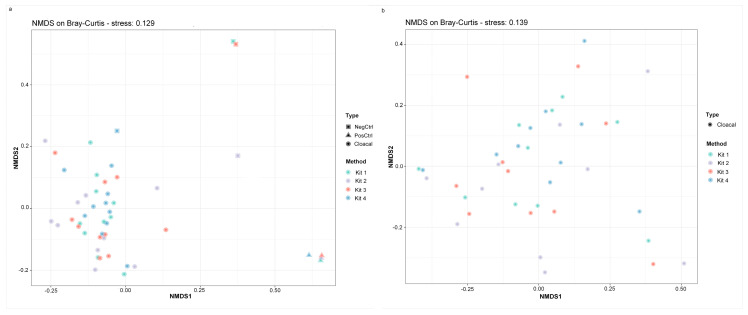
DNA extraction method does not affect bacterial community structure. Non-metric multidimensional scaling (NMDS) on Bray–Curtis distances with (**a**) and without (**b**) controls. The DNA extraction methods are shown with different colors and the type of sample (cloacal sample, negative control, or positive control) are shown with different shapes. Differences in community structure were not significant between DNA extraction methods (*p* > 0.05, PERMANOVA).

Detectable DNA was measured in all four negative controls (NC), which underwent library preparation and sequencing. Reads obtained from each NC were derived from 21 (Method 1), 36 (Method 2), 26 (Method 3), and 77 (Method 4) genera. The contaminant profiles of Method 1 and Method 3 NC were similar, but Method 2 and Method 4 were vastly different (Figure 2b). *Asinibacterium* and *Pseudomonas* were the most abundant genera detected in the NC of Method 1 and Method 2. Method 3 also had *Asinibacterium* as the most abundant genus, followed by unclassified taxa. *Burkholderia-Caballeronia-Paraburkholderia* and *Lactobacillus* dominated the bacterial composition of NC in Method 2. NMDS visualization showed that the negative controls of Method 1 and Method 3 clustered together and separately from the samples, while the negative control of Method 4 clustered closely to the cloacal samples (Figure 3a).

### 3.4. Taxonomic Profile and Community Diversity

**Figure 4:**
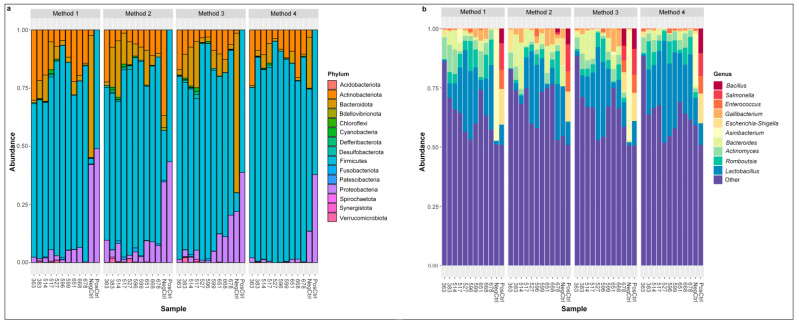
Distribution of the most abundant phyla and genera is similar across each DNA extraction method. Relative abundance of most prevalent phyla (**a**) and 10 most abundant genera (**b**) detected in cloacal samples among each DNA extraction method, where “Other” makes up the total relative abundance of other common genera.

The composition and the relative abundance of the bacterial communities detected in cloacal samples are presented in Figure 4. Firmicutes was the most abundant taxa at phylum level, followed by Actinobacteriota, Proteobacteria, and Bacteroidota (Figure 4a). One phylum had differences in relative abundance between methods after adjusting *p* values for multiple comparisons; Proteobacteria had the lowest relative abundance in Method 4 compared to Method 1 (*p* = 0.01, Dunn’s post hoc test), Method 2 (*p* < 0.001, Dunn’s post hoc test), and Method 3 (*p* = 0.001, Dunn’s post hoc test). The genus *Lactobacillus* was the most abundant taxa, followed by *Actinomyces, Bacteroides, Romboutsia, Enterococcus, Corynebacterium,* and *Gallibacterium,* and *Lactobacillus*, (Figure 4b). There were no differences in the relative abundance of any genera between methods.

**Figure 5:**
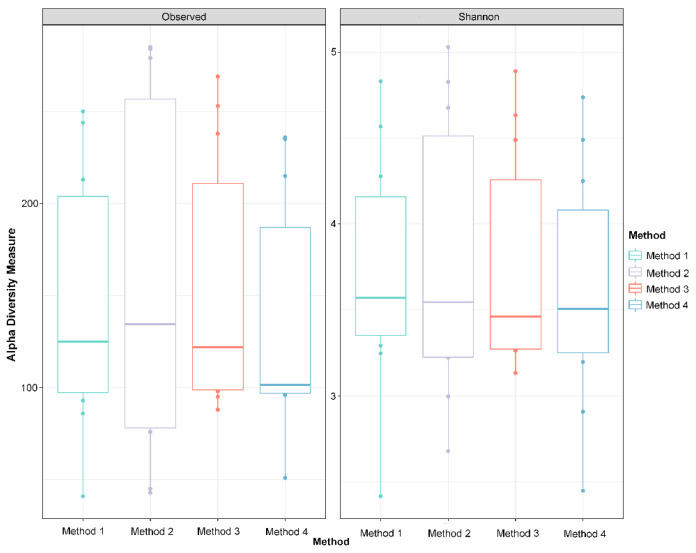
Alpha diversity indices do not differ between DNA extraction methods. Observed ASVs and Shannon’s Index, two metrics of alpha diversity, are visualized with boxplots where the middle bar represents the median, the boxes represent the interquartile range, and the whiskers extend to 1.58 times the interquartile range. There are no significant differences between DNA extraction methods in either diversity metric (*p* > 0.05, Kruskal–Wallis test).

Alpha diversity metrics, including observed taxa and Shannon’s diversity, were measured in R using dada 2. No significant differences among DNA extraction methods in either index were detected (Figure 5, Shannon’s diversity *p =* 0.4373; observed taxa *p* = 0.4372, Kruskal–Wallis test). Non-multidimensional scaling based on Bray–Curtis distances was performed to evaluate the impact of extraction method on microbial community composition (Figure 3). Cloacal samples clustered differently than the negative and positive controls (known community standard), except for the negative control from Method 4 (Figure 3a). The Bray–Curtis group dispersions were similar between methods (permutest *p* > 0.05), and therefore, beta diversity was tested with permutational ANOVA. Permutational ANOVA was performed both with and without the negative and positive controls, and no significant differences were detected between DNA extraction methods (with controls PERMANOVA R^2^ = 0.044, *p =* 0.89; without controls PERMANOVA R^2^ = 0.067, *p =* 0.59). Similar results were observed in the principal coordinate analysis (PCoA) based on weighted Unifrac distance (without controls PERMANOVA R^2^ = 0.062, *p =* 0.64) (Figure S2).

## 4. Discussion

Four commercial DNA extraction methods were tested on 10 cloacal samples from White Leghorn hens to assess if the extraction method affected library preparation or 16S sequencing results. We found that although the extracted DNA quality and quantity differed between methods, overall 16S sequencing results (alpha and beta diversity) were not affected, and only one phylum had differences in relative abundances between methods. Methods 3 and 4 (both protocols with the in-house bead loading system) resulted in higher DNA extraction yield with similar quality.

Differences in DNA yield among extraction methods had no effects on microbial diversity. This result is in accordance with studies using human feces and pig feces and hospital sewage [20,21]. Interestingly, some previous work in permafrost samples and soil samples concluded that the choice of DNA extraction method affected diversity analyses [10,11]. Altogether, this indicates that the impact of DNA extraction method on 16S diversity analyses is system-specific, which highlights the need to perform these comparisons across sample types.

Taxonomic analysis of cloacal samples showed similar composition at phylum and genus level despite extraction method and were similar to previous work in poultry. One phyla, Proteobacteria, was found to be significantly less relatively abundant in Method 4 than the three other extraction methods; however, there were no differences in genera. Firmicutes, Proteobacteria, and Actinobacteria have been found to be the most abundant phyla in cloacal samples in broiler and laying hens [7,22,23,24,25]. The most abundant genus across all samples was *Lactobacillus,* which has been previously described in young broiler chicks [25], but other groups have found that *Pseudomonas* and *Romboutsia* are the most abundant genera in laying hen cloacal swabs [7,23]. The chickens used in this study were randomly subset from a larger study on supplementation with metformin, which may result in these differences, in addition to environmental factors, such as diet, housing conditions, and geography [23,26]. Since we did not make comparisons between chickens, these do not drive the major findings of our experiment.

Mock communities and negative controls were also evaluated for each extraction method. The four positive controls generally agreed with the expected mock community values. However, it is important to note that contaminant genera were detected in all four positive controls that were not part of the expected mock community makeup, which were composed of *Faecalicoccus* and *Enterobacteriaceae*. With only one control per method, statistical testing was not possible, and there were only slight numerical differences in the relative abundance values for each bacterial strain compared to the expected mock community. Interestingly, *Lactobacillus* and *Escherichia-Shigella* were both also detected in the negative controls of all four methods, which suggests that these bacteria may be present as mock community standards as well as contaminants. In Method 2 and Method 4, *Lactobacillus* made up a relatively large percentage (~10%) of the total relative abundance of the negative controls.

Several dozen bacterial genera were detected in each of the four negative controls and differed between methods. The negative control of each method comprised the method’s included buffers that were added to a sample well at the beginning of each DNA extraction and carried through DNA extraction, amplification, library preparation, and 16S sequencing. As only one negative control was sequenced for each of the four methods, statistical hypothesis testing is not possible; however, there were wide numerical differences in both the total number of contaminant genera detected and the relative abundance of the most common genera between each method. These findings suggest that there is general laboratory contamination, as well as method-specific reagent contamination. *Asinibacterium* was the most common genera found in Methods 1, 3, and 4, while most contaminants in Method 2 were comprised of the least abundant genera (“Other” in Figure 3b) followed by *Bradyrhizobium*. The overall contaminant profiles were most similar between Method 1 and Method 3, while Method 2 and Method 4 had very different relative abundance profiles. The overall contaminant profiles were most similar between Method 1 and Method 3, while Method 2 and Method 4 had very different relative abundance profiles. Different contaminants were detected in the positive and negative controls in each commercial extraction method. Negative controls are expected to have lower bacterial biomass than samples and should, therefore, have fewer sequencing reads than experimental samples [27]. This was confirmed in our results, which found that the negative controls in all four methods had between ~4000 and ~50,000 reads. While having lower biomass, all negative controls yielded a substantial amount of data upon sequencing, reinforcing the need to include these controls in microbiome work and account for these contaminants in the analysis.

This study is limited by the relatively small sample size (*n* = 10 samples per method) although each method was compared in the same 10 animals, eliminating a potential source of intra-animal variability. Additionally, four methods from two commercially available kits were measured, and many more commercially available options exist for DNA extraction. Future research should compare additional methods (e.g., the Qiagen PowerSoil kit) to determine if those affect sequencing outcomes. The negative and positive controls were added at the extraction steps, and there were no unused swabs as an environmental negative control, so it is not possible to determine if any bacteria in the cloacal samples were environmental contaminants.

Previous research has indicated that the cloacal microbiome differs from the intestinal microbiome from cecal samples in chickens [6,7], and recent work has argued against the use of cloacal swabs to analyze the avian gut microbiome [24]. However, cecal or intestinal sampling currently requires euthanasia of the bird, which is not practical when performing longitudinal studies or researching in agricultural settings. Therefore, it is important to highlight that although it has shortcomings, cloacal swabs are the most appropriate choice of sampling for these study designs in birds where euthanasia is not feasible. Nevertheless, other studies have concluded that the cloacal microbiome is reasonably similar to the cecal microbiome in broiler chickens to allow for inferences [22]. It should also be noted that gastrointestinal anatomy can differ by bird species and sampling wild birds is subject to different challenges and complications than sampling broiler or laying chickens in agricultural settings; as this study analyzed cloacal samples from laying hens, we refrain from making further generalizations to different bird species. Perhaps most importantly, the research question should be considered when choosing the host sample location, which will aid in determining whether euthanasia is required to sample the intestine or ceca or if cloacal swabs or fecal samples will suffice. Further research is needed to assess the similarities of the microbial profiles of cloacal swabs, droppings, and different intestinal sections of birds for robust conclusions to be drawn.

## 5. Conclusions

Although both nucleic acid purification methods (Methods 3 and 4) had the highest extraction DNA yield and comparable extraction quality, the Method using Reagent DX (Method 4) resulted in the highest number of contaminant genera detected in the negative control and the lowest relative abundance of Proteobacteria in the cloacal swab samples. Therefore, it may be most appropriate to use clarifying solution in place of Reagent DX for DNA extraction in cloacal swabs. As no differences were observed in bacterial diversity metrics, we can conclude that the choice of specific DNA extraction method tested in this study does not affect overall sequencing outcomes in cloacal swabs; however, it is critically important to add both positive and negative extraction controls to microbiome projects in order to properly detect and account for contaminant bacteria. This research provides guidance to investigators in future studies on animal microbiomes, particularly those involving poultry cloacal samples.

## Figures and Tables

**Table 1:** The quality and quantity of DNA yield depends on the commercial DNA extraction method. Data are presented as median ± inter-quartile range. Lowercase letters indicate significant differences in DNA quantity between methods (post hoc Dunn test *p* < 0.05, *n* = 10/method). Asterisks indicate significance from the “ideal” ratio (Wilcox signed rank test *p* < 0.05, *n* = 10/method). No statistical analyses were performed on the A260/280 or A260/230 ratios.

	Quantity (ng/μL)	A260/280	A260/280 Difference from Ideal 1.8	A260/230	A260/230 Difference from Ideal 2.0
**Method 1**	3.77 ± 4.93 ^a^	2.00 ± 0.02	0.196± 0.20	0.085 ± 0.09	−1.92 ± 0.09 *
**Method 2**	5.87 ± 6.43 ^a^	1.67 ± 0.29	−0.13 ± 0.29 *	0.64 ± 0.42	−1.36 ± 0.42 *
**Method 3**	11.0 ± 3.94 ^b^	1.80 ± 0.06	0.001 ± 0.06	0.07 ± 0.03	−1.93 ± 0.03 *
**Method 4**	9.94 ± 7.71 ^b^	1.67 ± 0.22	−0.129 ± 0.22 *	0.04 ± 0.01	−1.96 ± 0.01 *

**Table 2:** Output read statistics of filtering and trimming using dada2 did not differ among the commercial DNA extraction method. Cloacal samples, positive, and negative controls were processed in the same way. Data are presented as median ± inter-quartile range. There were no significant differences between DNA extraction methods (*p* > 0.05, Kruskal–Wallis test).

Parameters	Method 1	Method 2	Method 3	Method 4	*p*-Value
**Input reads**	170,634 ± 15,754	163,742 ± 24,679	165,514 ± 30,082	175,852 ± 169,75	0.677
**Filtered reads**	156,912 ± 15,843	148,520 ± 28,729	151,676 ± 31,168	163,609 ± 17,997	0.444
**Merged reads**	143,496 ± 16,470	139,377 ± 26,720	141,729 ± 31,022	141,626 ± 20,923	0.488
**Non-chimeric reads**	124,625 ± 21,441	127,622 ± 28,734	109,974 ± 38,361	134,731 ± 24,383	0.549

## Data Availability

Raw data, including NanoDrop and Qubit values and R scripts, are deposited at https://github.com/gandalab/bird-dna-extraction. Sequencing reads are available at the NCBI Sequence Read Archive under accession number PRJNA720437.

## Supplementary Materials

The following are available online at https://www.mdpi.com/article/10.3390/ani11051372/s1, Table S1: Filtering parameters in dada2. Figure S1: A230/260 ratios following purification and PCR amplification, Figure S2: PCoA of weighted Unifrac distances.
