# Supplementary material for: Choice of Commercial DNA Extraction Method Does Not Affect 16S Sequencing Outcomes in Cloacal Swabs
Source: Animals (Basel). 2021 May 12;11(5):1372. doi: 10.3390/ani11051372 (PMC8151189; doi:10.3390/ani11051372)

Supplementary Materials

Table S1: Filtering parameters in dada2. MultiQC visualization indicated that kits differed in sequence quality, so a variety of filtering and trimming parameters were tested to determine the pipeline that optimized quality and read retention. Filtering and trimming parameters that were tested in dada2 are shown below, and asterisks represent the parameters chosen for data analysis.

Test	truncLen	trimLeft	maxN	maxE	truncQ	rm.phix	compress
**A**	c(290,280)	c(19,20)	2	2.5	2	TRUE	TRUE
**B**	c(290,280)	c(19,20)	2	2.2	2	TRUE	TRUE
**C**	c(300,280)	c(19,20)	2	2.5	2	TRUE	TRUE
**D**	c(300,280)	c(19,20)	2	2.2	2	TRUE	TRUE
**E**	c(280,280)	c(19,20)	2	2.5	2	TRUE	TRUE
**F**	c(280,280)	c(19,20)	2	2.2	2	TRUE	TRUE
**G**	c(290,290)	c(19,20)	2	2.5	2	TRUE	TRUE
**H**	c(290,290)	c(19,20)	2	2.2	2	TRUE	TRUE
**I**	c(300,290)	c(19,20)	2	2.5	2	TRUE	TRUE
**J**	c(300,290)	c(19,20)	2	2.2	2	TRUE	TRUE
**K***	c(275,280)	c(20,20)	2	2.5	2	TRUE	TRUE
**L**	c(275,280)	c(20,20)	2	2.2	2	TRUE	TRUE

Figure S1: A230/260 ratios following purification and PCR amplification. After further purification and PCR amplification for 16S primers, the A260/230 ratios are recovered to acceptable values. Extracted DNA was subjected to PCR and AmPure cleaning and NanoDrop was used to measure DNA quantity and quality. After cleaning, only Kit 2 had significant differences in the A230/260 ratio compared to the ideal 2.00 value (shown by asterisk; $p < 0.05$ Wilcox rank-sum test).

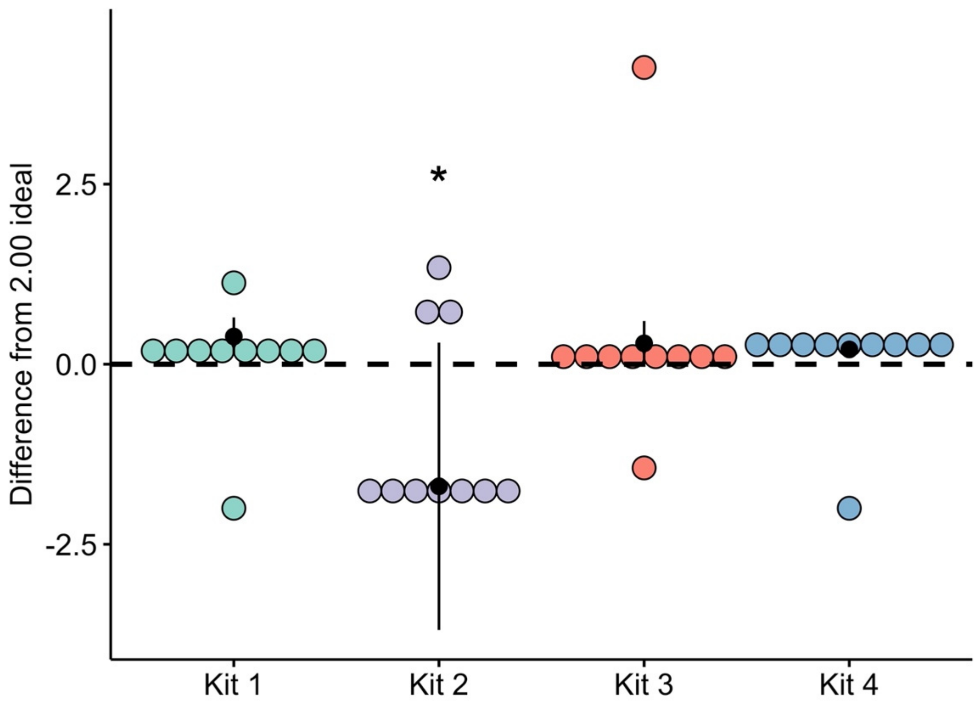

Figure S2: PCoA of weighted Unifrac distances. Bacterial beta diversity does not differ between DNA **extraction kits.** Principal coordinate analysis based on weighted Unifrac distance using data. Cloacal samples are shown in points (controls not shown). No significant differences were observed (PERMANOVA $R^2 = 0.062$, $p = 0.64$).

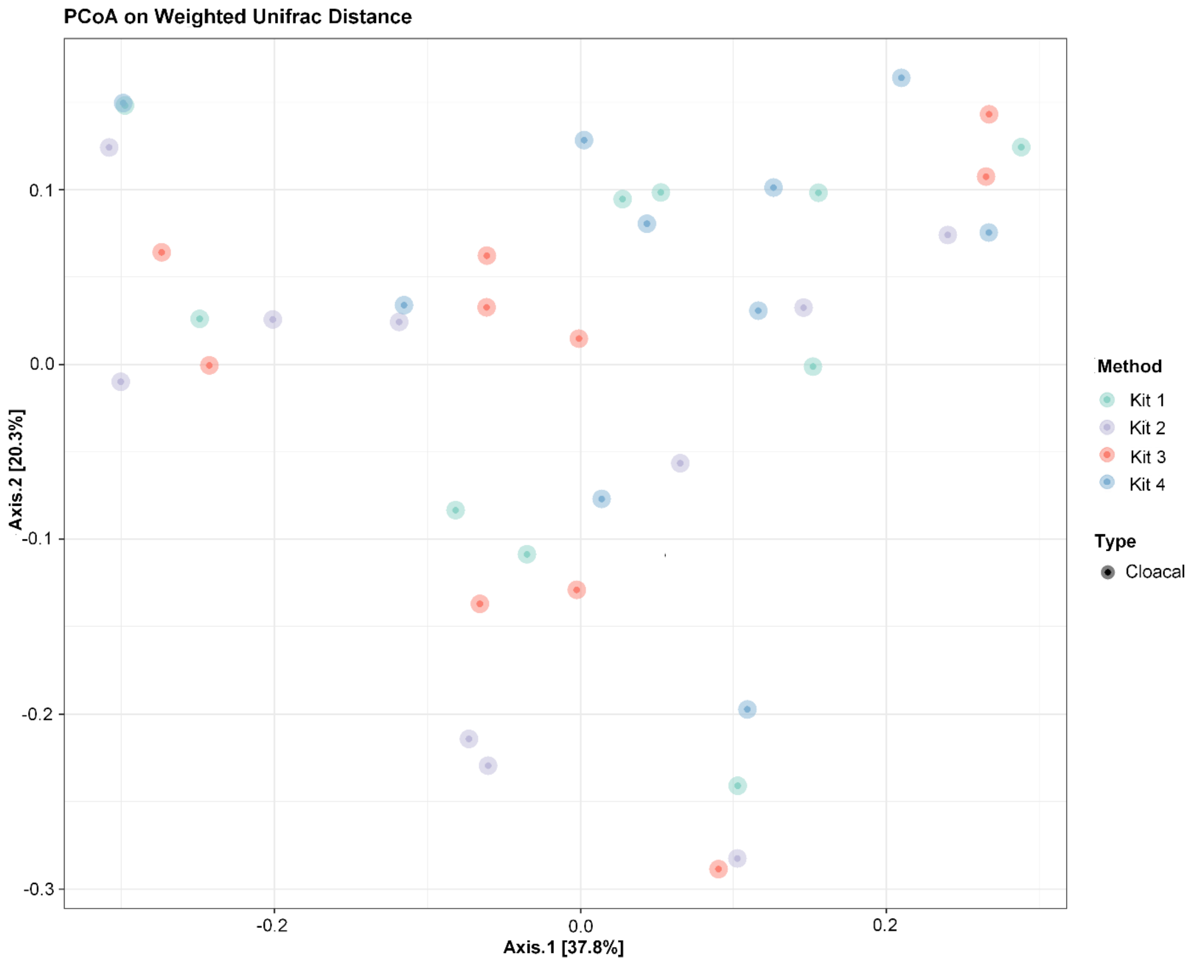

Supplement: Supplementary file 1 [file animals-11-01372-s001.zip › animals-1166689-supplementary.pdf]
